# Flora of Bokor National Park V: Two new species of *Machilus* (Lauraceae), *M. bokorensis* and *M. brevipaniculata*

**DOI:** 10.3897/phytokeys.65.7403

**Published:** 2016-06-15

**Authors:** Tetsukazu Yahara, Shuichiro Tagane, Keiko Mase, Phourin Chhang, Hironori Toyama

**Affiliations:** 1 Center for Asian Conservation Ecology, Kyushu University, 744 Motooka, Fukuoka, 819-0395, Japan; 2 Institute of Forest and Wildlife Research and Development, Forestry Administration, 40 Preah Norodom Blvd, Phnom Phen, Cambodia

**Keywords:** Bokor National Park, Cambodia, Lauraceae, Machilus, new species

## Abstract

Two new species, *Machilus bokorensis* Yahara & Tagane and *Machilus brevipaniculata* Yahara & Tagane (Lauraceae) are described from Bokor National Park, Cambodia with their illustrations and DNA barcodes of the two plastid regions of *rbcL* and *matK* and the nuclear region of ITS.

## Introduction

Lauraceae is a large family containing 54 genera and 2500–3500 species distributed from tropical to temperate areas in the world (Rohwer 1993). In Southeast Asia, species of Lauraceae are found from lowlands to high elevations, and are often dominant components of tropical and subtropical evergreen forests. Thus, some efforts have been made to elucidate species taxonomy of Lauraceae in Southeast Asia (e.g., Lecomte 1914; Liou 1934; Kostermans 1974, 1988; Kochummen 1989), including regional revisions for *Beilschmiedia* of Borneo (Nishida 2008), *Cinnamomum* of Borneo (Wuu-Kuang et al. 2011), *Cryptocarya* of Thailand and Indochina (de Kok et al. 2015) and *Litsea* of Thailand (Ngernsaengsuruay et al. 2011). However, no volumes on Lauraceae have been published in Flore du Cambodge du Laos et du Vietnam, Flora Malesiana, or Flora of Thailand. Thus, species of Lauraceae still remain difficult to identify in many areas of Southeast Asia.

The genus *Machilus* Nees is a member of the monophyletic *Persea* group consisting of *Alseodaphne* Nees, *Apollonias* Nees, *Dehaasia* Blume, *Machilus*, *Nothaphoebe* Blume, *Persea* Mill., and *Phoebe* Nees (Rohwer et al. 2009; Li et al. 2011). Although Kostermans (1990) merged *Machilus* into *Persea*, recent molecular evidence showed that *Persea* of the New World may be polyphyletic and *Machilus* of Asia is well differentiated from the two clades including the majority of the Neotropical *Persea* species (Rohwer et al. 2009; Li et al. 2011). *Machilus* comprises about 100 species in tropical and subtropical S and SE Asia (Wei and van der Werff 2008). Twelve *Machilus* species (including *Machilus balansae* (Airy Shaw) F.N.Wei & S.C.Tang, *Machilus cochinchinensis* Lecomte, and *Machilus velutina* Champ. ex Benth., all treated as *Persea*) are recorded from Vietnam (Hô 1999), three species from Laos (Newman et al. 2007), eight species from Malaysia (treated as *Persea*, Kochummen 1989), and 82 species from China (Wei and van der Werff 2008). In Cambodia, only one species, *Machilus odoratissima* Nees, has been reported based on specimens (Lecomte 1914; Liou 1934; Phon 2000); three additional species, *Machilus gamblei* King ex Hook.f., *Machilus salicina* Hance, and *Machilus velutina*, were recorded from Cambodia in Flora of China (Wei and van der Werff 2008), but we could not find any specimens supporting those records.

During our field surveys of vascular plants in Bokor National Park, Kampot Province, Southern Cambodia conducted from 2011 to 2013, we collected five species of the genus *Machilus* among which two fertile species which differ from all known congeners. Here we describe them as new, *Machilus bokorensis* Yahara & Tagane and *Machilus brevipaniculata* Yahara & Tagane, with illustrations and DNA barcodes of the two plastid regions of *rbcL* and *matK* (CBOL Plant Working Group 2009), and the nuclear ITS region.

The specimens examined are deposited in the herbarium of Forest Administration of Cambodia, Phnom Penh (not in Index Herbariorum, abbreviated ‘PNP’ here), the Herbarium of the Museum of Kyushu University
(FU) and partly in the Herbarium of the Kyoto University Museum
(KYO) and the Forest Herbarium, Bangkok (BKF).

## Materials and methods

### Morphological observations

We have made morphological observations on the specimens that we had collected in Bokor National Park, Cambodia. We have surveyed the diagnostic features of all the known species in Cambodia and its neighboring regions by the means of a thorough literature review and by morphological observations of the dry specimens, kept in the herbaria BKF, BO, FU, HN, K, KYO, L, MBK, P, RAF, SAR and VNM and specimen images available on the web [e.g. JSTOR Global Plants (https://plants.jstor.org/)].

### DNA barcoding

Pieces of leaves were collected and desiccated with silica gel in the field. DNA was extracted from these samples using a modified CTAB method. Before DNA extraction, we ground silica gel-dried leaves with a TissueLyser (QIAGEN) into the powder that were subsequently washed at least four times with 1 mL buffer (0.1 M HEPES, pH 8.0; 2% mercaptoetanol; 1% PVP; 0.05 M ascorbic acid). We determined the partial sequences of DNA regions encoding the large subunit of ribulose-1,5-bisphosphate carboxylase oxygenase (*rbcL*) and maturase K (*matK*), following the published protocols (Kress et al. 2009; Dunning and Savolainen 2010). Additionally, we determined the sequences of the internal transcribed spacer (ITS) region using the primers of ITS18-F (5’-GTCCACTGAACCTTATCATTTAGAGG-3’) and ITS26-R (5’-GCCGTTACTAAGGGAATCCTTGTTAG-3’) according to the published protocol (Rohwer et al. 2009) with slight modification using Tks GflexTM DNA Polymerase (TAKARA, Japan).

## Taxonomic treatments

### Machilus bokorensis

Taxon classificationPlantaeLauralesLauraceae

Yahara & Tagane sp. nov.

urn:lsid:ipni.org:names:77155497-1

Figs 1 , 2

Machilus odoratissima (non Nees) sensu H.Liou, Laurac. Chine & Indochine: 53 (1932), for the specimen collected from Nord de Kampot (P, no. 14625).

#### Diagnosis.

Similar to *Machilus odoratissima* Nees in elliptic-lanceolate or oblanceolate, glabrous leaves and subterminal inflorescences, but differing in having sericeous inflorescence rachis (Fig. 2c; vs. glabrous in *Machilus odoratissima*), perianth lobes pubescent on both surfaces (Fig. 2c; vs. outside almost glabrous), ca. 1 mm long staminodes (vs. ca. 2 mm long), and twigs drying blackish grey (vs. reddish). Similar to *Machilus parviflora* Meisn. among Indochinese species in having large terminal buds, but differing in bud scales densely puberulent outside (vs. densely golden hairy outside in *Machilus parviflora*).

**Figure 1.**
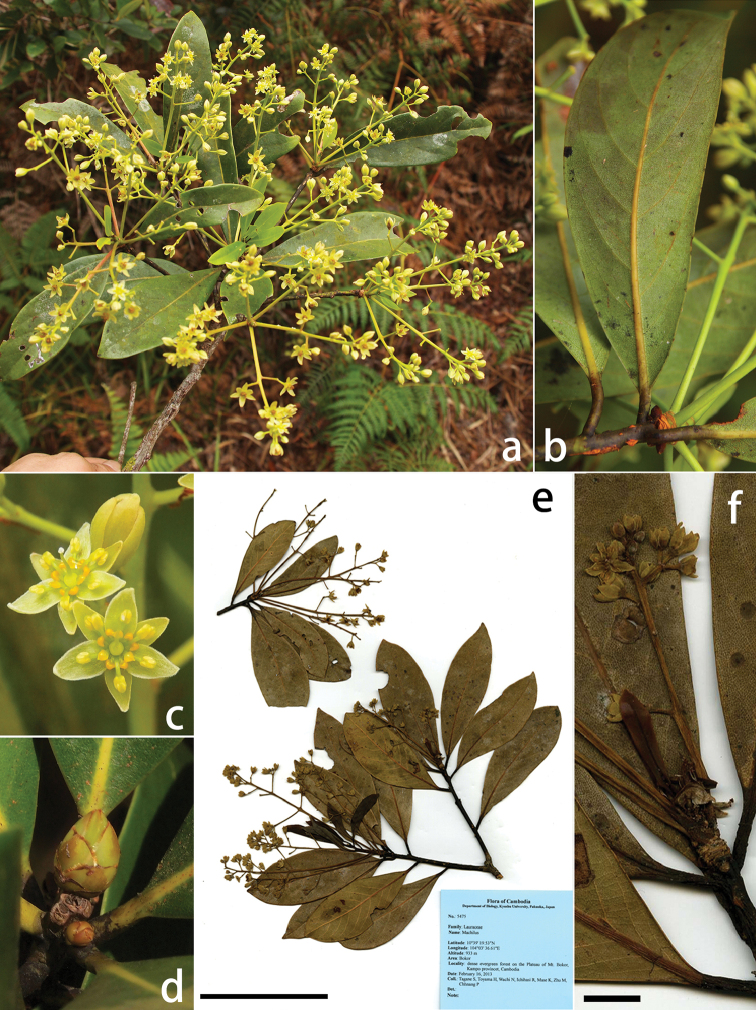
*Machilus bokorensis* Yahara & Tagane. **a** flowering branch, **b** abaxial lower surface of lamina **c** flowers **d** buds **e** holotype: *Tagane et al. 5475* (KYO), scale bar = 10 cm **f** inflorescence, scale bar = 1 cm. Photographs: **a–c** by S. Tagane, **d** by K. Fuse.

**Figure 2.**
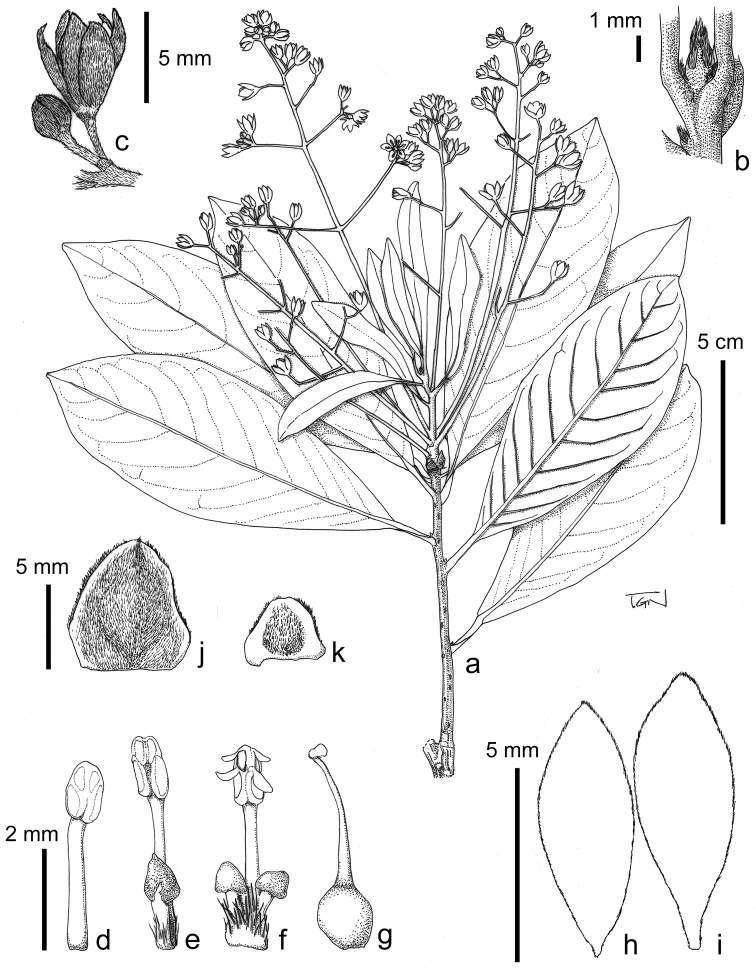
*Machilus bokorensis* Yahara & Tagane. **a** flowering branch **b** top of branch **c** flowers **d** stamen in 1st whorl **e** stamen in 2nd whorl with staminode **f** stamen in 3rd whorl with glands **g** pistil **h** outer perianth lobes **i** inner perianth lobe **j, k** bud scales. Materials from *Tagane et al. 5475* (KYO). Drawn by S. Tagane.

#### Type.

CAMBODIA. Kampot Province, Bokor National Park, dense evergreen forest on the plateau of Mt. Bokor, 10°39'19.5"N, 104°03'36.6"E, alt. 933 m, 16 Feb. 2013, with flowers, *Tagane S., Toyama H., Wachi N., Ichihasi R., Mase K., Zhu M., Chhnang P. 5475* (holotype KYO!, isotypes BKF!, FU!, K, P, PNP!).

#### Description.

Small tree, 10 m tall. Branchlets glabrous, blackish when dry, sparsely lenticellate, old branches blackish grey or blackish grey brown. Terminal buds 3–7 mm long, bud scales broadly ovate to oblanceolate, densely puberulent outside, glabrous or very sparsely pubescent inside. Leaves alternate, blades elliptic-lanceolate, oblanceolate, (3–)7.5–11.5(–18.5) × (1.2–)2.5–4.0(–5.4) cm, thickly leathery, base cuneate, margin entire, slightly revolute, apex usually acute to obtuse, occasionally acuminate or rotund, developing leaves glabrous adaxially, sericeous abaxially, glabrous and densely foveolate on both sides when old, midrib concave adaxially, elevated abaxially, lateral veins 9–12 pairs, flat adaxially, slightly prominent abaxially, tertiary veins reticulate to scalariform-reticulate; petioles (0.5–)0.8–1.3(–2.5) cm long, glabrous. Inflorescences paniculate, subterminal, usually arising from near base of newly sprouted branchlets, (4.0–)6.0–13.0 cm long, sericeous, with 5–8 lateral branches. Pedicels 3–5 mm long, sericeous, bracts linear, 1–4 mm long, abaxially yellowish brown pubescent, adaxially glabrous, caducous. Flowers yellowish green; perianth lobes 6, oblong-elliptic, slightly unequal, outer ones, ca. 6.8 × 2.4 mm, inner ones ca. 7 × 2.5 mm, minutely pubescent on both surfaces. Stamens 9, subequal, 3.5–4 mm long, hairy at base, glands of 3rd series stipitate, ca. 0.4 mm long; staminodes 3, ca. 1 mm long, stipitate, glabrous. Ovary subglobose ca. 1.5 mm in diam., glabrous. Style 2.5–3 mm long, glabrous. Fruits subglobose, 7–8 mm in diam., glabrous, blackish when dry, perianth lobes spreading.

#### Ohter specimens examined.

Cambodia, Bokor National Park [alt. 1043 m, 10°37'16.8"N, 104°01'52.3"E, 10 Dec. 2013, *Toyama et al. 6258* (BKF, FU, PNP); alt. 1042 m, 10°38"N, 104°01.4’E, 9 Mar. 2001, with fruits, *Middleton & Monyrak 643* (P); alt. 1014 m, 10°38'12.6"N, 104°02'06.4"E, 4 Dec. 2011, *Toyama et al. 1556* (FU, PNP); ibidem, *Toyama et al. 1575* (FU, PNP); alt. 1011 m, 10°38'11.2"N, 104°02'09.0"E, *Toyama et al. 1725* (FU, PNP); alt. 928 m, 10°39'19.5"N, 104°03'36.6"E, *Toyama et al. 2754* (FU, PNP); alt. 903 m, 10°39'35.4"N, 104°03'03.1"E, *Toyama et al. 3167* (FU, PNP); alt. 871 m, 10°39'48.8"N, 104°02'53.3"E, 17 Dec. 2013, with flowers, *Fuse et al. 6227* (BKF, FU, PNP)].

#### Distribution.

Cambodia (endemic to Mt. Bokor).

#### Habitat and ecology.

*Machilus bokorensis* is commonly found in moist evergreen forest on the top plateau of Mt. Bokor. Flowering specimens were collected in December and February and fruiting specimens in March.

#### GenBank accession No.

*Toyama et al. 1575*: AB987667 (*rbcL*), AB987666 (*matK*), AB987665 (ITS).

#### Preliminary conservation assessment.

*Machilus bokorensis* is commonly found in higher elevations in Mt. Bokor. Since many mature individuals were found in the protected areas, we suggest the status for this species as Least Concern (LC) according to IUCN Red List criteria (IUCN 2012).

#### Note.

Among five species we collected in Mt. Bokor, this species is most common and agrees with the description of “*Machilus odoratissima*” in the key of Liou (1934) who recorded “*Machilus odoratissima*” from Nord de Kampot, corresponding to Mt. Bokor. Thus, we consider that this species is “*Machilus odoratissima*” recorded by Liou (1934), although we could not examine the specimen of *Poilane 14625* (P) cited by Liou (1934) because it is currently on loan. In Indo-china, *Machilus* plants similar to this species have been identified as *Machilus odoratissima* for a long time (Lecomte 1914; Liou 1934; Phon 2000). However, *Machilus bokorensis* is distinct from *Machilus odoratissima* distributed in Nepal, E Himalaya, Assam-Burma in sericeous inflorescence rachis (vs. glabrous in *Machilus odoratissima*; Pendry 2012), perianth lobes pubescent on both surfaces (vs. outside almost glabrous), ca. 1 mm staminodes (vs. ca. 2 mm), and blackish grey twigs (vs. reddish). In Cambodia, we collected sterile specimens of *Machilus* spp. morphologically similar to *Machilus bokorensis* in Central Cardamon and Seima protection forests but those are morphologically distinguishable and not sister to *Machilus bokorensis* in our unpublished phylogenetic tree constructed by *rbcL*, *matK* and ITS sequences. “*Machilus odoratissima*” is recorded from Mulu-prey, Preah Vihear (Lecomte 1914). The specimens from Mulu-prey (*Harmand s.n.*, P) are, however, different from *Machilus bokorensis* in having naked terminal buds and scalariform tertiary veins. Lecomte (1914) and Liou (1934) recorded “*Machilus odoratissima*” also from Laos and Vietnam, but we have not found specimens from Laos and Vietnam identical with *Machilus odoratissima*. As far as we know, *Machilus odoratissima* is not distributed in Cambodia, Laos, Thailand and Vietnam. The relationship between *Machilus bokorensis* and the specimens of Mulu-prey, Laos and Vietnam remains to be further examined. At present, we consider that *Machilus bokorensis* is endemic to Bokor, Cambodia. According to our unpublished ITS tree, this species is sister to *Machilus rimosa* (Blume) Blume distributed in Indonesia, but distinct from *Machilus rimosa* in thickly leathery leaves densely foveolate on both sides (vs. thinner leaves not densely foveolate in *Machilus rimosa*) and perianths larger (6.8–7 mm long vs. ca. 3.5 mm long).

### Machilus brevipaniculata

Taxon classificationPlantaeLauralesLauraceae

Yahara & Tagane sp. nov.

urn:lsid:ipni.org:names:77155498-1

Figs 3 , 4

#### Diagnosis.

Similar to *Machilus kingii* Hook.f. in leaf shape and size, and short panicles less than 5.5 cm long, but distinct from *Machilus kingii* by its naked bud (vs. bud covered with scales in *Machilus kingii*), lamina foveolate on both surfaces (vs. obscure on lower surface), fewer flowers per panicle (3–5 vs. 4–9), and smaller perianth lobes (2 mm vs. 3–3.5 mm long).

#### Type.

CAMBODIA. Kampot Province, semi-evergreen secondary forest at the bottom of Mt. Bokor, 10°35'35.6"N, 103°58'43.1"E, alt. 65 m, 7 Dec. 2013, *Tagane S., Toyama H., Fuse K., Iwanaga F., Rueangruea S., Suddee S., Kanemitsu H., Zhang M., Kim W., Loth M. 6011* (holotype KYO!, isotypes BKF!, FU!, PNP!).

#### Description.

Small tree, 8 m tall. Branchlets sericeous near the tip, soon glabrous, brownish when dry, old branches greyish brown to reddish brown, without lenticels; terminal buds naked. Leaves alternate; blades lanceolate to oblanceolate, 5.0–8.3 × 1.4–3.1 cm, leathery, reddish brown when dried, glabrous and foveolate on both sides, lustrous adaxially when dry, base cuneate, margin slightly revolute when dry, apex acute to obtuse, midrib concave adaxially, elevated abaxially, lateral veins 8–10 pairs; petioles 0.5–1.4 cm, glabrous. Inflorescences paniculate, terminal or arising as lateral branches of newly sprouted shoots, 4–11 cm long, usually with frondose bracts subtending secondary paniculate axes, secondary axes 0.8–1.3 cm long with 3–15 flowers. Pedicels ca. 2 mm long. Flowers yellowish green. Outer perianth lobes ovate, ca. 2 × 1 mm, inner ones broadly ovate, ca. 2 × 1.3 mm, pubescent on both surfaces, with many gland dots. Stamens 9, subequal, ca. 1.5 mm long, hairy at base, anthers 4-celled, ca. 1 mm long; glands of 3rd series stipitate, ca. 0.3 mm diam. Staminodes 3, ca. 0.6–1 mm long, glabrous. Ovary ellipsoidal, ca. 0.8 mm in diam., glabrous. Style ca. 0.8 mm long, glabrous. Fruits not seen.

**Figure 3.**
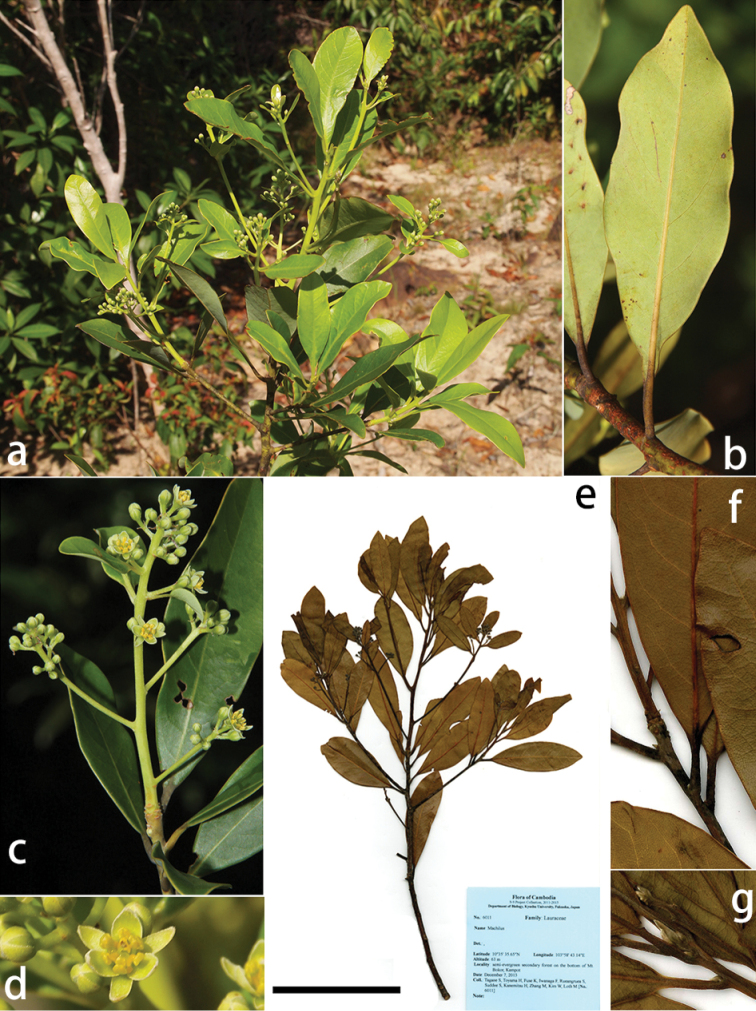
*Machilus brevipaniculata* Yahara & Tagane. **a** flowering branch **b** flowers **c** abaxial lower surface of lamina **d** flower **e** holotype: *Tagane et al. 6011* (KYO), scale bar = 10 cm, **f** portion of leaves **g** apical bud. Photographs: **a**, **b**, **d** by S. Tagane and **c** by S. Rueangruea.

**Figure 4.**
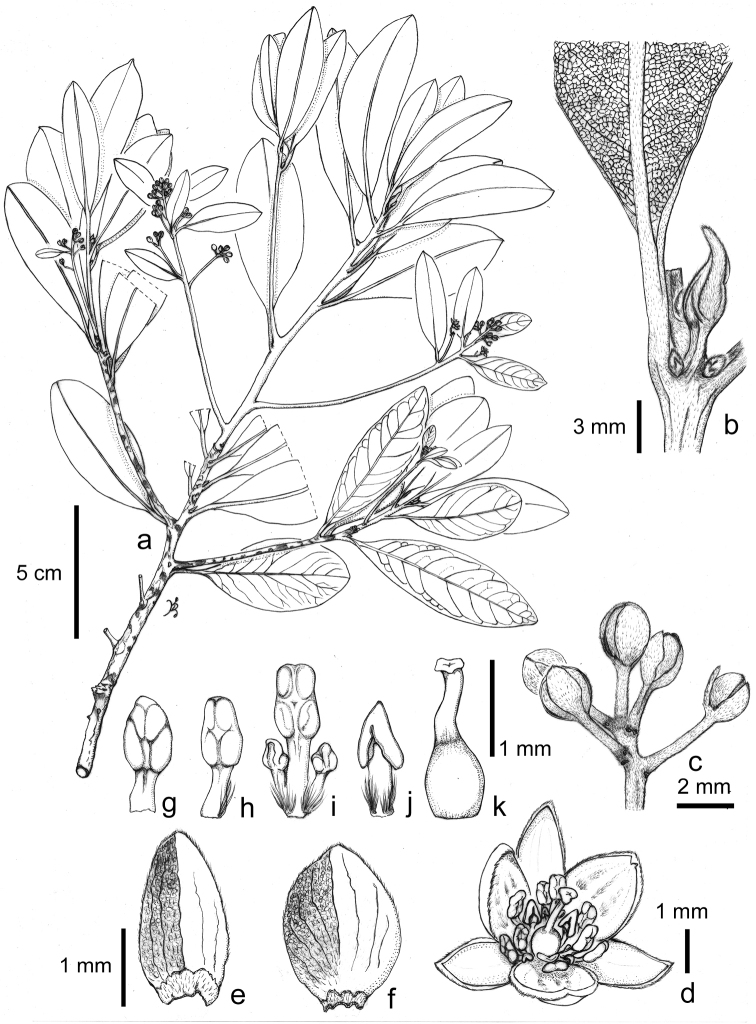
*Machilus brevipaniculata* Yahara & Tagane, **a** flowering branch **b** top of branch with abaxial leaf surface **c** flower buds **d** flower **e** outer perianth lobe **f** inner perianth lobe **g**–**i** stamens in 1st, 2nd and 3rd whorl from left **j** staminode **k** pistil. Materials from *Tagane et al. 6011* (KYO). Drawn by K. Mase.

#### Distribution.

Cambodia. Known only from the lowland of Bokor National Park.

#### Habitat and ecology.

This species was found in evergreen forest at the foot of Mt. Bokor. Flowering specimens were collected in December.

#### GenBank accession No.

*Tagane et al. 6011*: AB987676 (*rbcL*), AB987675 (*matK*), AB987674 (ITS).

#### Preliminary conservation assessment.


 Critically endangered (CR) (IUCN 2012). We collected only one flowering individual of this species at alt. 65 m during 7 field surveys on the southern slope and top plateau of Mt. Bokor (Tagane et al. 2015). Further botanical inventories might enable us to find more individuals, but it seems to be a rare species. Considering the fact that the forest is almost cleared in the foot of Mt. Bokor (below 100 m in particular), we believe that this species should be considered critically endangered.

#### Note.

This species is distinct in having naked terminal buds and panicles having frondose bracts subtending secondary paniculate axes, while most of *Machilus* species have scaly terminal buds and panicles without frondose bracts. According to our unpublished ITS tree, this species is sister to *Machilus coriacea* A.Chev. endemic to southern Vietnam, but can be readily distinguished from the latter by the two above mentioned traits and also in having glabrous leaves (vs. densely hairy beneath when young in *Machilus coriacea*).

## Supplementary Material

XML Treatment for Machilus bokorensis

XML Treatment for Machilus brevipaniculata
